# The 2021 Dutch Working Party on Antibiotic Policy (SWAB) guidelines for empirical antibacterial therapy of sepsis in adults

**DOI:** 10.1186/s12879-022-07653-3

**Published:** 2022-08-11

**Authors:** Elske Sieswerda, Hannelore I. Bax, Jacobien J. Hoogerwerf, Mark G. J. de Boer, Marja Boermeester, Marc J. M. Bonten, Douwe Dekker, Roy Gerth van Wijk, Nicole P. Juffermans, Marnix Kuindersma, Paul D. van der Linden, Damian C. Melles, Peter Pickkers, Jeroen A. Schouten, Jasper R. Rebel, Arthur R. H. van Zanten, Jan M. Prins, W. Joost Wiersinga

**Affiliations:** 1 Department of Medical Microbiology, University Medical Centre Utrecht, Utrecht University, Utrecht, The Netherlands; 2 Department of Internal Medicine, Section of Infectious Diseases and Department of Medical Microbiology and Infectious Diseases, Erasmus University Medical Center, Rotterdam, The Netherlands; 3 Department of Medicine, Radboud Center for Infectious Diseases, Radboud University Medical Center, Nijmegen, The Netherlands; 4 Department of Infectious Diseases, Leiden University Medical Center, Leiden, The Netherlands; 5 Department of Surgery, Amsterdam UMC, University of Amsterdam, Amsterdam, The Netherlands; 6 Julius Center for Health Sciences and Primary Care, University Medical Center Utrecht, Utrecht University, Utrecht, The Netherlands; 7 Department of Internal Medicine, University Medical Centre Utrecht, Utrecht, The Netherlands; 8 Division of Allergology, Department of Internal Medicine, Erasmus Medical Center, Rotterdam, The Netherlands; 9 Department of Intensive Care, OLVG Hospital, Amsterdam, The Netherlands; 10 Department of Intensive Care, Gelre ziekenhuis Apeldoorn, Apeldoorn, The Netherlands; 11 Department of Clinical Pharmacy, Tergooi Hospital, Hilversum, The Netherlands; 12 Department of Medical Microbiology and Immunology, Meander Medical Center, Amersfoort, The Netherlands; 13 Department of Intensive Care Medicine, Radboud Center for Infectious Diseases, Radboud University Medical Center, Nijmegen, The Netherlands; 14 Department of Emergency Medicine, OLVG Hospital, Amsterdam, The Netherlands; 15 Department of Intensive Care Medicine, Ziekenhuis Gelderse Vallei, Ede, The Netherlands; 16 Division of Infectious Diseases, Department of Medicine, Amsterdam UMC, University of Amsterdam, Amsterdam, The Netherlands

**Keywords:** Antibacterial therapy, Duration of antibiotic therapy, Sepsis, Guidelines, Empirical therapy, Pharmacokinetics, Pharmacodynamics, Enterobacterales, *Pseudomonas aeruginosa*, Antimicrobial resistance, Penicillin allergy

## Abstract

**Background:**

The Dutch Working Party on Antibiotic Policy (SWAB) in collaboration with relevant professional societies, has updated their evidence-based guidelines on empiric antibacterial therapy of sepsis in adults.

**Methods:**

Our multidisciplinary guideline committee generated ten population, intervention, comparison, and outcome (PICO) questions relevant for adult patients with sepsis. For each question, a literature search was performed to obtain the best available evidence and assessed using the Grading of Recommendations Assessment, Development, and Evaluation (GRADE) system. The quality of evidence for clinically relevant outcomes was graded from high to very low. In structured consensus meetings, the committee formulated recommendations as strong or weak. When evidence could not be obtained, recommendations were provided based on expert opinion and experience (good practice statements).

**Results:**

Fifty-five recommendations on the antibacterial therapy of sepsis were generated. Recommendations on empiric antibacterial therapy choices were differentiated for sepsis according to the source of infection, the potential causative pathogen and its resistance pattern. One important revision was the distinction between low, increased and high risk of infection with Enterobacterales resistant to third generation cephalosporins (3GRC-E) to guide the choice of empirical therapy. Other new topics included empirical antibacterial therapy in patients with a reported penicillin allergy and the role of pharmacokinetics and pharmacodynamics to guide dosing in sepsis. We also established recommendations on timing and duration of antibacterial treatment.

**Conclusions:**

Our multidisciplinary committee formulated evidence-based recommendations for the empiric antibacterial therapy of adults with sepsis in The Netherlands.

**Supplementary Information:**

The online version contains supplementary material available at 10.1186/s12879-022-07653-3.

## Background

Sepsis is currently defined as life-threatening organ dysfunction caused by a dysregulated host response to infection [1–4]. Sepsis and septic shock are common reasons for intensive care unit (ICU) admission and associated with high mortality rates, even at long-term follow-up [5–12]. Worldwide in 2017, nearly 50 million cases of sepsis were recorded resulting in 11 million sepsis-related deaths [13]. In the Netherlands the estimated annual number of admissions for severe sepsis in Dutch ICU’s was 7700–9500 in 2004 [14]. The incidence of sepsis may have risen in recent decennia, possibly due to ageing and increasing numbers of immunocompromised patients [6, 8, 15]. Antibacterial treatment is an essential part of effective sepsis management. Inappropriate or delayed antibacterial treatment in patients with sepsis and septic shock have been associated with increased morbidity and mortality [16–21].

The Dutch Working Party on Antibiotic Policy (SWAB), initiated by the Dutch Association of Internal Medicine, the Dutch Society for Medical Microbiology and the Dutch Association of Hospital Pharmacists, coordinates activities in the Netherlands intending to optimize antibiotic use, to contain the development of antimicrobial resistance, and to limit the costs of antibiotic use. The general objective of the SWAB sepsis guidelines is to guide medical professionals in the empirical antibacterial treatment for adults with sepsis and septic shock in hospitals. The current guidelines on empirical antibacterial therapy of sepsis in the Netherlands is an update of the SWAB sepsis guidelines published in 2010 [22].

Providing evidence-based recommendations on empirical antibacterial therapy in sepsis is challenging. There is considerable heterogeneity among sepsis studies as to included patients (comorbidities, disease severity, source of infection), microbiological characteristics (availability of culture results, pathogens involved, local antimicrobial resistance), interventions (drug dosing, source control, timing of treatment) as well as to the outcome parameters assessed. In particular, antimicrobial resistance is much lower in the Netherlands than in other countries [23–25]. Another important consideration is that most trials and meta-analyses on antibacterial therapy are not powered to assess relevant outcomes such as adverse events and the development of antimicrobial resistance [26–28].

In this publication, we summarize the most important literature and changes in recommendations for the antibacterial treatment of adults with sepsis.

## Methods

For a complete description of the methodology of the guideline, we refer to the original document. In short, the guideline was written according to the Appraisal of Guidelines for Research and Evaluation (AGREE II) instrument [29].

A multidisciplinary guideline committee consisting of experts delegated from relevant professional societies followed a guideline development process comparable to that of the Infectious Diseases Society of America (IDSA), which includes a systematic method of grading both the quality of evidence (very low, low, moderate, and high) and the strength of the recommendation (weak or strong) [30]. We aimed to provide an overview of the quality of available evidence and give evidence-based recommendations for antibacterial treatment of sepsis in adults (≥ 18 years old). We restricted the guideline to the most important causes of sepsis, i.e., pneumonia, abdominal infections, urinary tract infections, complicated skin and soft tissue infections, as well as to sepsis in general or of (yet) unknown origin. Neutropenic sepsis, sepsis due to viral or fungal infections, sepsis in patients with prosthetic material or long term central venous catheters, sepsis due to osteomyelitis, meningitis, mediastinitis and endocarditis and children were outside the scope of the guideline.

The committee generated ten population, intervention, comparison, and outcomes (PICO) questions relevant for adult patients with sepsis in the Netherlands (Table 1). For each question we reviewed existing national and international guidelines and performed additional pragmatic literature searches. For evidence on drug resistance in the Netherlands, the guideline committee used surveillance data from 2017 in the NethMap annual report 2018 [23]. Reports of the European Committee on Antimicrobial Susceptibility Testing (EUCAST) guided the interpretation of susceptibility test results [31].

**Table 1:** Key questions SWAB guideline for empirical antibacterial therapy of sepsis in adults

I Causative bacterial pathogens in sepsis
1 Which bacteria are most frequently isolated from patients with sepsis in the Netherlands?
2 What are the resistance patterns of the most frequently isolated bacteria in patients with sepsis in the Netherlands?
3 Which patients are at risk for sepsis due to third-generation cephalosporin-resistant Enterobacterales (3GCR-E) or *P. aeruginosa* in the Netherlands?
II Empirical antibacterial therapy of sepsis
4 What is the importance of appropriate empirical therapy in patients with sepsis?
5 What is the effect of double active empirical antibacterial therapy compared to monotherapy in patients with sepsis?
6 What is the optimal choice of empirical therapy in patients with sepsis in the Netherlands?
7 What is the optimal empirical antibacterial therapy of sepsis in patients with a penicillin allergy?
III Timing and duration of antibacterial therapy in sepsis
8 What is the optimal timing of empirical antibacterial therapy in patients with sepsis?
9 What is the optimal duration of antibacterial treatment for sepsis?
IV Pharmacokinetic and pharmacodynamic considerations in sepsis
10 In patients with sepsis, should we recommend pharmacokinetic/pharmacodynamic dosing optimization for empirical antibacterial therapy?

Included guidelines and studies were assessed using the Grading of Recommendations Assessment, Development, and Evaluation (GRADE) system. We graded the quality of evidence for clinically relevant outcomes from high to very low. In structured consensus meetings, the committee formulated recommendations as strong or weak. When evidence could not be obtained, recommendations could be provided based on opinions and experiences (good practice statements).

The draft guideline was submitted to the members of relevant professional societies for external review. The guideline working group adjusted the guideline according to comments in the external review through group discussion. Both comments and responses of the committee are available at www.swab.nl. The final version received formal approval from the SWAB executive board.

## Results

### Causative bacterial pathogens in sepsis and their antibiotic susceptibility

#### Which bacteria are most frequently isolated from patients with sepsis in The Netherlands?

In the Netherlands, the most commonly cultured pathogens in blood cultures are coagulase-negative staphylococci (CNS) (34%), *Escherichia coli* (23%), *Staphylococcus aureus* (10%)*, Klebsiella pneumonia (4%)* and *Enterococcus faecalis/faecium* (5%) [23]. In patients with sepsis and ICU admission, gram-negative pathogens were more likely to be involved [32, 33]. Of note, *Acinetobacter baumannii* was not an important cause of sepsis due to hospital-acquired pneumonia (HAP) or ventilator-associated pneumonia (VAP) as it was hardly isolated in respiratory cultures of hospitalized patients in Dutch surveillance data [23]. Reported pathogens in sepsis due to intra-abdominal infections were *E. coli*, enteric anaerobes, other Enterobacterales, *Enterococcus* spp. and *Streptococcus* spp. [34]. In central line-associated bloodstream infections (CLABSI), the most reported pathogens were CNS, gram-negative bacteria (fermenters and non-fermenters), *S. aureus*, *Enterococcus* spp. and *Candida albicans* [35, 36].

#### What are the resistance patterns of the most frequently isolated bacteria in patients with sepsis in The Netherlands?

A Dutch study among 648 intensive care unit (ICU) patients with non-pneumonia derived sepsis reported microbiological culture results of (surveillance) samples obtained two days before until two days after ICU admission. Resistance percentages of pathogenic bacteria in these patients were 10% for 3rd generation cephalosporins, 8% for ciprofloxacin, 6% for gentamicin, 2% for piperacillin-tazobactam, and 0.5% for meropenem [37]. Dutch surveillance data showed that the rate of extended-spectrum beta-lactamase (ESBL)-producing bacteria in blood cultures has increased over the past years. In 2017, 6% of *E. coli* and 10% of *K. pneumoniae* blood isolates were resistant to 3rd generation cephalosporins (Table 2) [23]. The prevalence of carbapenem resistance in all *E. coli* and *K. pneumoniae* isolates was stable over five years and low at 0.03% and 0.42%. The risk of methicillin-resistant *S. aureus* (MRSA) bacteraemia has remained stable over the last ten years and low at 1% of all *S. aureus* bacteraemias [23].

**Table 2:** Percentage of growth and resistance of most frequent pathogens in blood cultures of patients in unselected departments in the Netherlands in 2017 [23]

	In blood culture	Amoxicillin-clavulanic acid	Cefuroxime	Ceftriaxone	Gentamicin	Ciprofloxacin	Piperacillin-tazobactam	Amoxi-clav + gentamicin	Amoxi-clav + ciprofloxacin	Cefuroxime + gentamicin	Cefuroxime + ciprofloxacin	Ceftriaxone + gentamicin	Ceftriaxone + ciprofloxacin
*E. coli*	23%	37%	12%	6%	4%	14%	5%	3%	9%	2%	6%	1%	4%
*K. pneumoniae*	4%	17%	14%	10%	5%	14%	7%	4%	9%	4%	9%	4%	7%
*P. mirabilis*	1%	8%	1%	1%	5%	11%	1%	2%	2%	0%	0%	0%	0%
*E. cloacae*	1%				3%	5%							
Other Enterobacterales	5%												
*P. aeruginosa*	2%				2%	9%	5%						
*S. aureus*	10%	1%		1%	0%	6%	1%						
Other Gram-positives	12%												

#### Which patients are at risk for sepsis due to third-generation cephalosporin-resistant Enterobacterales (3GCR-E) or *Pseudomonas aeruginosa* in the Netherlands?

One systematic review summarized colonization and risk of subsequent bacteraemia with ESBL-producing Enterobacterales in patients with solid and haematological malignancies [38]. Patients with known colonization with an ESBL-producing Enterobacterales as detected by surveillance cultures (mostly at admission) were 13 times more likely to develop a bacteraemia with these pathogens compared to patients not that were not colonized. Two specific risk factors for sepsis due to 3GCR-E have been externally validated by in a Dutch retrospective study of 9442 episodes in which blood cultures were drawn and iv antibacterial therapy was started [39]. Positive predictive values (PPV) of prior (90 days and 1 year) colonization with 3GCR-E were 7.4% and 6.1% for predicting bacteraemia and 34.4% and 28.2% for predicting any culture-positive infection with 3GCR-E. PPVs of prior (30 days) treatment with cephalosporins or fluoroquinolones were 1.3% for predicting bacteraemia and 6.9% for predicting any culture-positive infection with 3GCR-E. No other studies were found that externally validated predictors for sepsis due to 3GCR-E or *P. aeruginosa*.

Based on currently available evidence, we concluded that prior (1 year) infection or colonization is the strongest and most common risk factor predicting subsequent infection with 3GCR-E [38, 40–42]. It was challenging to provide general recommendations on other risk factors that should be taken into account to guide the decision to start empirical antibiotic therapy in sepsis directed against 3GCR-E or *P. aeruginosa*. Until high-quality and externally validated prediction rules are available, the guideline committee recommends that the following factors should be taken into account to decide if empirical antibacterial therapy against 3GCR-E in patients with sepsis is appropriate: local prevalence of 3GCR-E [43], whether the sepsis is hospital-acquired [40, 44, 45], and to a lesser extent healthcare-associated, versus community-acquired, whether the patient received prior (2 months) antibiotic treatment and whether or not the patient receives selective decontamination of the digestive tract (SDD) [40, 45, 46]. It is essential to realize the limitations of using risk factors for the decision to treat for 3GCR-E, to weigh potential risk factors against the associated risk of overtreatment and to ensure antibiotic de-escalation if possible.

In addition, the committee regarded the high colonization rate with highly resistant micro-organisms (HRMO) in travellers from highly endemic countries such as the Indian subcontinent as another risk factor to consider in the choice of empirical antibiotic therapy in patients with sepsis. As many travellers will not be colonized anymore after several months, we suggest including three months prior travel to highly endemic countries in the individual risk assessment (https://resistancemap.cddep.org). The committee felt that the risk of 3GCR-E involvement to be high in patients with sepsis recently hospitalized abroad for > 24 h. There is no strong evidence to support this statement, but it is in accordance to national infection prevention guidelines on which patients to screen for HRMO [47]. We therefore included this as a separate suggestion.

Regarding *P. aeruginosa*, the committee suggests to empirically start targeted treatment in patients with sepsis when prior (1-year) cultures showed *P. aeruginosa*. In addition, we suggest covering *P. aeruginosa* in patients with sepsis due to HAP/VAP or suspected infected central venous catheter (CVC) infection.

### Empirical antibacterial therapy in sepsis

#### What is the importance of appropriate empirical therapy in patients with sepsis?

The importance of appropriate empirical antibacterial therapy in patients with sepsis has been supported by systematic reviews of observational studies only [21, 48, 49]. The reported effect has been consistent and includes reduced mortality, costs and length of hospital stay, although with considerable heterogeneity between studies [21, 48, 49]. Very low quality evidence showed a trend towards improved outcomes of appropriate empirical therapy in patients with sepsis due to HRMO and anaerobic pathogens [43, 50–52]. For *Enterococcus* spp, empirical treatment strategies in community-acquired intra-abdominal infections showed no difference in clinical outcomes comparing antibiotic regimens with and without activity against Enterococci [52]. There is no clear evidence to support or refute empirical treatment of enterococci in hospital-acquired intra-abdominal infections, patients that have no adequate source control, immunocompromised patients and patients with sepsis [52].

Based on the available evidence, the committee strongly recommends empirical broad-spectrum antibacterial therapy for patients presenting with sepsis to cover all pathogenic bacteria that are likely to be involved. Prior (< 1 year), relevant cultures and local distribution of pathogens associated with sepsis and their antimicrobial susceptibilities should guide the ultimate choice. Although there is a lack of strong evidence, the committee suggests to empirically cover HRMO when these are likely to be involved and to cover anaerobic bacteria in patients presenting with abdominal sepsis or necrotizing soft tissue infections. We suggest against the routine empirical treatment of anaerobic bacteria in sepsis due to aspiration pneumonia, unless empyema or a lung abscess is suspected. Similarly we recommend against the routine empirical treatment of enterococci, but to consider treatment in individual patients with sepsis, such as those who have a high likelihood of enterococcal involvement based on recent relevant cultures and those with recent complicated intra-abdominal surgery or a suspected CVC infection and substantial exposure to broad spectrum antibiotics.

#### What is the effect of double active empirical antibacterial therapy compared to monotherapy in patients with sepsis?

We defined double active antibacterial therapy as treatment with two classes of antibiotics, both targeting the known or suspected causing pathogen(s) (e.g., ceftriaxone and an aminoglycoside to target gram-negative pathogens) and with the specific purpose to accelerate pathogen clearance rather than to broaden antimicrobial coverage.

Pooled data in a meta-analysis showed no additional effect on all-cause mortality and clinical failure of beta-lactam plus aminoglycoside double active therapy compared to the same or a different beta-lactam when given as monotherapy in patients with sepsis [53]. An increased risk of clinical failure and nephrotoxicity for beta-lactam plus aminoglycoside double active therapy compared to a different beta-lactam given as monotherapy was reported [53]. Other meta-analyses and randomized trials also showed no additional effect of empirical double active antibacterial therapy compared to empirical monotherapy on clinical outcomes in patients with sepsis and septic shock [54], patients with *S. aureus* bacteraemia [55], patients with severe *P. aeruginosa* infections [53, 56, 57], and patients with VAP [58, 59].

Based on these data the committee recommends against the use of double active antibacterial therapy in patients with sepsis and septic shock, provided that the chosen single antibacterial agent is active against the most likely pathogens involved. In line, we suggest against double active antibacterial therapy in patients with sepsis due to *P. aeruginosa* and *S. aureus*.

#### What is the optimal choice of empirical therapy in patients with sepsis in The Netherlands

Most trials in patients with severe infections compared cephalosporins, carbapenems, piperacillin-tazobactam and some fluoroquinolones. Clinical outcomes did not consistently support that one of these classes of antibiotics is considerably more effective than others in patients with sepsis. Aminoglycoside-based regimens for sepsis due to HAP or VAP were associated with lower clinical response rates [59]. For sepsis due to intra-abdominal infections, aminoglycoside monotherapy was less effective compared to beta-lactam treatment [60, 61]. One large randomized multicentre trial (MERINO) compared definitive therapy with piperacillin-tazobactam to meropenem in patients with bloodstream infections caused by ceftriaxone-resistant, piperacillin-tazobactam and meropenem sensitive *E. coli* and *K. pneumonia* [62]. The 30-day all-cause mortality was 12.3% in patients treated with piperacillin-tazobactam and 3.7% in patients treated with meropenem. There were no trials available on optimal antibiotic treatment of sepsis and high likelihood of *S. aureus* involvement*.*

The guideline committee concluded that based on the current data about efficacy and safety of beta-lactams, the experience with beta-lactams and the large number of trials using a beta-lactam, beta-lactams are most appropriate as empirical and definite therapy in the majority of patients with sepsis. Based on the available literature, fluoroquinolones are acceptable alternatives when the risk of fluoroquinolone resistance is considered low. However, clinicians should be aware that the use of fluoroquinolones has significant disadvantages regarding toxicity and the development of resistance [63–66]. Regarding aminoglycosides, the committee expresses their concerns on potential lower efficacy and higher toxicity risk, but settled that current (lack of) evidence still supports *short-term* (i.e., maximum of 2 days) aminoglycoside treatment added to a beta-lactam agent in patients with sepsis with the only purpose of increasing the empirical antibacterial spectrum of activity until susceptibility results are available. This strategy is therefore mainly applicable to gram-negative bacteria such as 3GCR-E or *P. aeruginosa*. Although questions remain, the committee found the evidence on the difference in mortality in the MERINO trial convincing enough to currently recommend against the use of BL/BI and specifically piperacillin-tazobactam for the treatment of sepsis in patients at risk of or with proven involvement of 3GCR Enterobacterales [62].

**Fig. 1:**
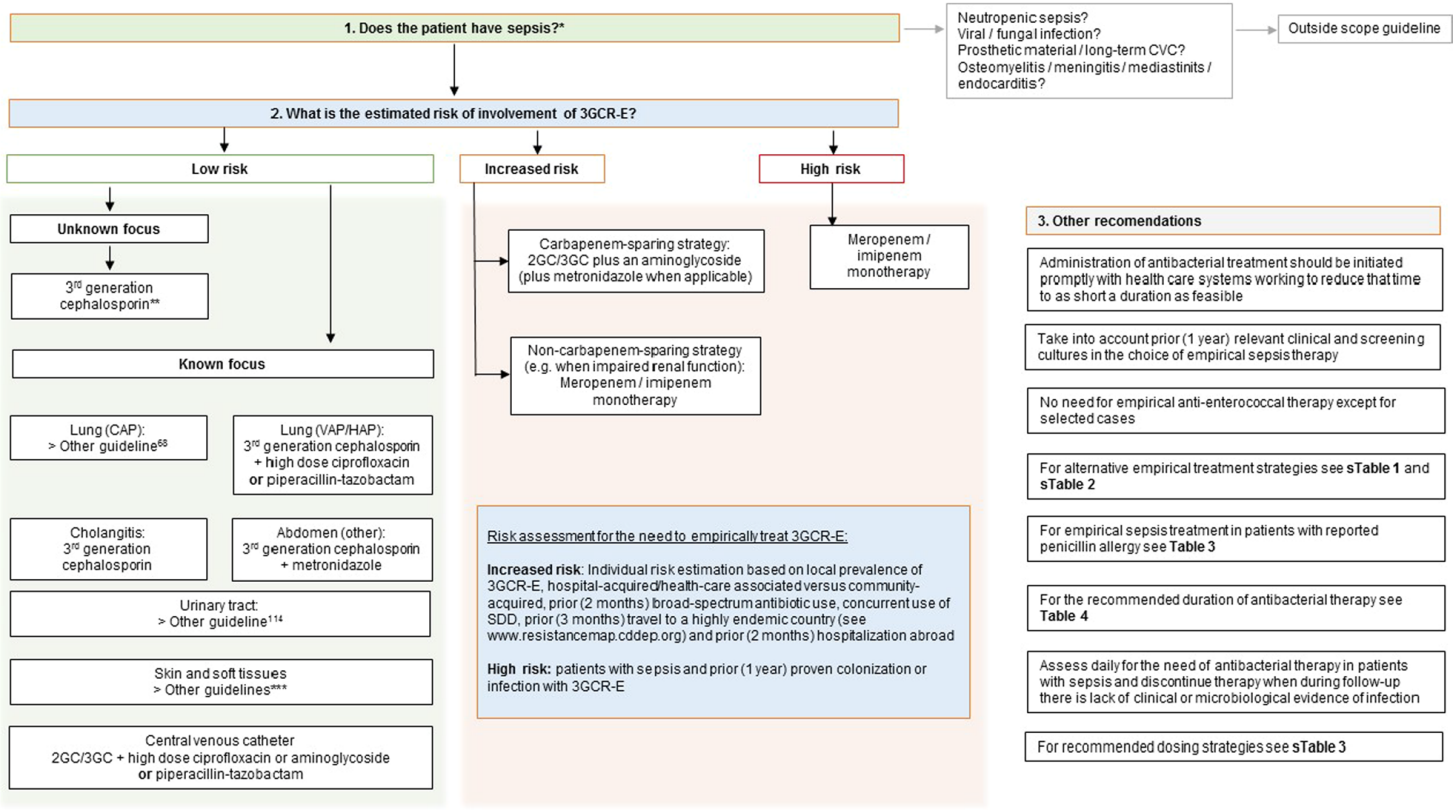
Flow chart of guideline recommendations on empirical antibiotic treatment of sepsis. *For the diagnosis and non-antibiotic treatment of sepsis we refer to the Dutch guideline ‘Sepsis fase 1’ [4]. **For this guideline, 3GC includes ceftriaxone and cefotaxime and does not include the anti-pseudomonal cephalosporin ceftazidime. ***Guidelines on skin and soft tissue infections [109, 110]. 3GCR-E: 3rd generation cephalosporin-resistant Enterobacterales; 2GC: second generation cephalosporin; 3GC: 3rd generation cephalosporin; SDD: selective decontamination of the digestive tract. CVC: central venous catheter; CAP: community-acquired pneumonia; VAP: ventilator-associated pneumonia; HAP: hospital-acquired pneumonia

The choice of empirical sepsis therapy is primarily based on the source of infection. Empirical treatment strategies should be further dictated by the likelihood of involvement of a resistant causative pathogen, by the desire to prevent overuse of reserve antibiotics from a stewardship perspective and by risks of toxicity and other potential adverse events for the patient. The committee therefore provided pragmatic suggestions and alternative strategies for patients with low risk of 3GCR-E involvement and patients at increased or high risk of involvement of 3GCR-E (Additional file 1: Tables S1 and S2). Recommendations are also summarized in Fig. 1. If a definite diagnosis is established one should be referred to other guidelines for empiric antibiotic therapy, e.g., current community-acquired pneumonia (CAP) guidelines do apply in the case of pneumonia-derived sepsis [67].

#### What is the optimal empirical antibacterial therapy of sepsis in patients with a penicillin allergy?

Accumulating data from observational cohort studies indicate that true penicillin allergy in patients with a reported penicillin allergy is relatively rare and that avoiding beta-lactams negatively affects treatment outcome [68, 69]. The committee set up a pragmatic approach based on available observational studies including a strong recommendation to obtain information (i.e., medical history and skin test results) about the presumed allergy if possible (Table 3).

**Table 3:** Empirical antibacterial therapy of sepsis in patients with a penicillin allergy label

Available allergy label data for penicillins (e.g., amoxicillin, amoxicillin-clavulanic acid, flucloxacillin, penicillin G)	Administration of a penicillin during sepsis	Administration of a cephalosporin or carbapenem during sepsis
Immediate type or delayed type^a^ reaction very unlikely	Yes	Yes
Possible immediate type reaction occurred > 10 years ago AND symptoms were mild to moderate	No^b^	Yes
Possible immediate type reaction occurred < 10 years ago AND/OR reaction was severe (i.e., anaphylactic shock, airway oedema etc.)	No^b^	Yes^c^
Allergy testing previously confirmed immediate type penicillin allergy	No	Yes^c^
Information about the allergy label is not available	No^b^	Yes

^a^In case of delayed type reactions e.g., Stevens-Johnson syndrome (SJS), toxic epidermic necrolysis (TEN), tubulointerstitial nephritis (TIN), on a beta-lactam antibiotic, avoid the respective penicillin and choose alternative treatment or consult expert; ^b^After the patient has recovered from sepsis, skin testing and/or controlled challenge with a beta-lactam may be considered to confirm or rule out allergy to beta-lactams; ^c^Risk of a severe immediate type cross allergic reaction is still estimated to be < 1%; Exposure may be avoided until skin-tests or controlled challenge is possible

### Timing and duration of antibacterial therapy in sepsis

#### What is the optimal timing of empirical antibacterial therapy in patients with sepsis?

In the previous edition of the SWAB sepsis guidelines, it was recommended to start antibacterial therapy in adult patients with severe sepsis and septic shock as soon as possible, preferably within the first hour of presentation [22]. The recommendation was mainly based on the results of one study showing that each hour delay in antibiotic therapy resulted in an average decrease in survival of 7.6% [19], an observation that was underlined by other retrospective observational studies [70–73]. However, a more recent meta-analysis, which included the aforementioned observational studies, did not show a significant mortality benefit of administering antibiotics within 3 h of ER triage or within 1 h of shock recognition in sepsis [74]. In line, a randomized trial on this topic could not demonstrate an effect of faster (prehospital) antibiotic administration for sepsis on outcome in a Dutch setting [32]. This study however only included a small number of patients with septic shock.

Based on available evidence, the committee deemed it reasonable to state that in patients with septic shock, antibiotics should be administered as soon as possible [71, 72]. On the other hand, in sepsis patients without shock, rapid antibiotic administration should be weighed against the negative impact of potentially unjustified antibiotic use when the patient turns out not to suffer from sepsis [75–77].

The guidelines committee therefore agreed not to recommend a specific timeframe in which antibiotics should be administered in patients with sepsis and septic shock. In line with a Dutch trial on the impact of emergency department staff training on time to antibiotic administration and with an earlier Infectious Disease Society of America (IDSA) position statement, the committee recommends that the administration of antibacterial treatment in patients with sepsis or septic shock should be initiated promptly with health care systems working to reduce that time to as short as feasible [32, 75].

#### What is the optimal duration of antibacterial treatment for sepsis?

Several meta-analyses [59, 78, 79], an RCT [80] and a large propensity-adjusted observational study [81] consistently showed that shorter duration of treatment is as effective and safe as the traditional, longer duration of treatment, in patient with sepsis. Similar results have been found in patients with mild to moderate-severe CAP [67], pyelonephritis [82], uncomplicated cellulitis [83], and bacteraemia [84]. In line, indirect evidence from the studies on PCT-guided discontinuation of antibacterial treatment in patients with sepsis in the ICU setting also suggests that shorter antibacterial treatment duration is safe without a negative effect on mortality [85–88]. These data, together with the potential adverse effects of antibiotic overuse, strengthened the committee to generally suggest durations of antibiotic therapy in most patients with sepsis that are shorter than historical treatment durations. Table 4 shows recommended treatment durations based on source of infection. Based on available evidence [85, 89–91], the committee suggests using PCT levels to support shortening the duration of antibacterial therapy in patients with sepsis if the optimal duration of antibiotic therapy is unclear.

**Table 4:** Suggested antibacterial therapy duration in patients with sepsis

Focus of sepsis	Suggested antibacterial treatment duration
Intra-abdominal infections, following effective source control and with favourable clinical response	Four days [111–114]
Cholangitis, following adequate drainage of the biliary tree	Up to 3 days [115]
VAP	Seven days [59]
HAP	Seven days
CVC infection with gram-negative pathogen, following removal of the CVC and with favourable clinical response	Up to 7 days [80]
CVC infection with CNS or enterococci, following removal of the CVC and with favourable clinical response	Zero to 7 days
No clear focus	Seven days [80]

Studies showed conflicting findings on the efficacy and safety of antibiotic de-escalation (ADE) [92–95]. Within the SWAB sepsis guideline committee there was consensus that ADE is appropriate in many clinical situations. In line with other relevant guidelines the committee recommends to consider ADE in all patients who are on sepsis treatment after 48 h of treatment [88, 96]. We also suggest this would include patients in whom only limited or indirect cultures show no causative pathogen. In contrast, with current conflicting evidence, including the negative outcomes of ADE in one trial on ICU length of stay the committee felt it is defendable not to perform ADE in selected individual patients [95].

### Pharmacokinetic and pharmacodynamic considerations in sepsis

#### In patients with sepsis, should we recommend pharmacokinetic/pharmacodynamic dosing optimization for empirical antibacterial therapy?

Many pathophysiological changes typical for sepsis patients can alter the pharmacokinetic properties of antibiotics and can lead to inadequate antibiotic concentrations when using standard antibiotic dosing schedules [97–102]. These pathophysiologic changes include kidney dysfunction, augmented renal clearing (the enhanced renal function sometimes seen in critically ill patients), hypoalbuminemia and increased third space due to fluid therapy [96, 98]. Drug concentrations of hydrophilic antibacterial agents (such as beta-lactams, aminoglycosides and vancomycin) are generally more sensitive to pharmacokinetic changes in patients with sepsis than lipophilic antibacterial agents (such as fluoroquinolones). In addition, patients with sepsis may generally be at higher risk to be infected with bacteria with higher MICs in comparison to other patients [98].

Pooled RCT data in patients with sepsis showed that extended or continuous infusion of beta-lactams in general was associated with decreased all-cause mortality, increased clinical cure with no effect on adverse events and development of resistance compared to intermittent infusion. Evidence was particularly strong for extended infusion of piperacillin-tazobactam and meropenem [103–105]. There was lack of evidence for the effect of pharmacokinetic/pharmacodynamic (PK/PD)-based dosing on clinical outcomes of aminoglycosides, vancomycin and ciprofloxacin and in obese patients. The committee felt that the available evidence supports a recommendation of PK/PD-based dosing [96, 98, 106–108]. Since EUCAST recommendations on breakpoints are generally accepted and based on PK/PD principles, we followed the EUCAST dosing recommendations for specific pathogens (Additional file 1: Table S3) [31]. We recommended therapeutic drug monitoring (TDM) for all patients on aminoglycoside and vancomycin treatment.

For a complete list of guidelines recommendations, see Table 5. A flow chart is provided in Fig. 1, which summarizes the given recommendations on the empirical antibacterial treatment of sepsis. See Text box 1 for a summary of all the new recommendations compared with the 2010 guideline. The full guidelines text, literature review and rebuttal of the received commentaries are available at www.swab.nl.

**Table 5:** Recommendations of the SWAB sepsis guideline 2021

Recommendation	Strength	Quality of evidence
I Causative bacterial pathogens in sepsis		
Which patients are at risk for sepsis due to third-generation cephalosporin-resistant Enterobacterales or *P. aeruginosa* in the Netherlands?		
1. We recommend empirical therapy against 3GCR-E in patients with sepsis and prior (1 year) proven infection or colonization with 3GCR-E	Strong	Very low
2. We suggest that clinicians take into account the risk of 3GCR-E involvement in sepsis on an individual patient basis to decide if empirical antibacterial therapy against 3GCR-E is appropriate Factors to guide this decision include local prevalence of 3GCR-E, if the infection is hospital-acquired/health-care associated versus community-acquired, prior (2 months) broad-spectrum antibiotic use, concurrent use of SDD, prior (3 months) travel to a highly endemic country (see https://resistancemap.cddep.org/) and prior (2 months) hospitalization abroad	Weak	Very low
3. We recommend empirical therapy against *P. aeruginosa* in patients with sepsis and prior (1 year) infection or colonization with *P. aeruginosa*	Strong	Very low
II Empirical antibacterial therapy in sepsis		
What is the importance of appropriate empirical therapy in patients with sepsis?		
4. We recommend empirical broad-spectrum antibacterial therapy for patients presenting with sepsis to cover all pathogenic bacteria that are likely to be involved	Strong	Moderate
5. We recommend to take into account prior (1 year) resistance in relevant clinical and screenings cultures in the choice of empirical sepsis therapy	Strong	Very low
6. We recommend that empirical antibacterial therapy is guided by the local distribution of pathogens associated with sepsis and their antimicrobial susceptibilities	Strong	Very low
7. We suggest empirical antibacterial therapy for patients presenting with sepsis to cover HRMO when these are likely to be involved	Weak	Very low
8. We suggest empirical antibacterial therapy covering anaerobic bacteria for patients presenting with sepsis and intra-abdominal infections of the lower intestinal tract or necrotizing soft tissue infections	Weak	Very low
9. We generally suggest against routine empirical treatment of anaerobic bacteria in patients presenting with sepsis due to aspiration pneumonia, unless empyema or a lung abscess is suspected	Weak	Very low
10. We generally recommend against routine empirical treatment of enterococci in patients presenting with sepsis	Strong	Moderate
11. We suggest that anti-enterococcal therapy could be considered in individual patients with sepsis, e.g., those who have a high likelihood of enterococcal involvement based on recent relevant cultures and those with recent complicated intra-abdominal surgery or a suspected CVC infection and substantial exposure to broad spectrum antibiotics	Weak	Very low
What is the effect of double active empirical antibacterial therapy compared to monotherapy in patients with sepsis?		
12. We recommend against routine double active empirical antibacterial therapy^a^ for patients with sepsis or septic shock	Strong	Moderate
13. We suggest that double active therapy is not routinely used as definite therapy for patients with sepsis due to *P. aeruginosa* infection	Weak	Very low
14. We suggest that double active therapy is not routinely used as definite therapy for patients with sepsis due to *S. aureus* infection not associated to prosthetic material	Weak	Moderate
What is the optimal choice of empirical therapy in patients with sepsis in the Netherlands?		
* Antibacterial therapy in patients with sepsis in general*		
15. In patients with sepsis, we generally recommend using a beta-lactam antibiotic covering the most likely involved pathogens	Strong	Moderate
16. In patients with sepsis in general / with no obvious source of infection, we suggest a 3rd generation cephalosporin (3GC). Alternative empirical treatment strategies are listed in Additional file 1: Table S1	Weak	Low
17. In patients with sepsis due to HAP or VAP, we suggest that there are equivalent empirical treatment strategies, listed in Additional file 1: Table S1	Weak	Low
18. In patients with sepsis due to cholangitis, we suggest a 3GC. Alternative empirical treatment strategies are listed in Additional file 1: Table S1	Weak	Low
19. In patients with sepsis due to intra-abdominal infection, we suggest a combination of a 3GC with metronidazole Alternative empirical treatment strategies are listed in Additional file 1: Table S1	Weak	Low
20. In patients with sepsis and a suspected CVC infection^b^, we recommend prompt removal of the line	Strong	GPS
21. In patients with sepsis and suspected CVC infection, we suggest empirical treatment with a 3GC^c^ with gentamicin or high dose ciprofloxacin Alternative treatment strategies are listed in Additional file 1: Table S1	Weak	GPS
22. For the empirical treatment of sepsis due to UTI, CAP and SSSI’s, we refer to other guidelines [67, 109, 110, 116]		
* Antibacterial therapy in patients with sepsis and increased risk of involvement of 3GCR-E*		
23. In patients with sepsis and high risk of involvement of 3GCR-E based on prior (1 year) infection/colonization, we recommend meropenem or imipenem as empirical antibacterial therapy Alternative strategies are listed in Additional file 1: Table S2	Strong	Moderate
24. In patients with sepsis and increased risk of involvement of 3GCR-E but no prior (1 year) infection/colonization, we suggest that a carbapenem-sparing strategy Additional file 1: Table S2 is acceptable	Weak	Very low
25. We cannot provide a recommendation for or against empirical or definite treatment with piperacillin-tazobactam in patients with sepsis due to chromosomal AmpC-producing Enterobacterales (such as *Enterobacter, Serratia, Citrobacter, Providencia* and *Morganella* spp)	–	–
26. In patients with sepsis due to ESBL-producing Enterobacterales, we recommend against piperacillin-tazobactam as definite antibacterial therapy regardless of the in vitro susceptibility	Strong	Moderate
*Antibacterial therapy in patients with sepsis and increased risk of involvement of Staphylococcus aureus*		
27. There is insufficient evidence to recommend against empirical use of other beta-lactam antibiotics than flucloxacillin or cefazolin in patients with sepsis in which *S. aureus* is a likely pathogen	-	-
28. For definite therapy of patients with sepsis due to *S. aureus*, we refer to the Dutch guideline on *S. aureus* bacteraemia [117]		
What is the optimal empirical antibacterial therapy of sepsis in patients with a penicillin allergy?		
29. In patients with sepsis and a reported penicillin allergy, we recommend to obtain information (i.e., medical history and skin test results) about the presumed allergy if possible	Strong	GPS
30. In patients with sepsis and a reported penicillin allergy but in whom the allergy is very unlikely, we suggest that penicillins can be used if needed (see Table 3)	Weak	Very low
31. In patients with sepsis and a reported penicillin allergy that was proven, possible or unspecified, we suggest to avoid penicillins during the primary sepsis treatment and to choose alternative beta-lactams (cephalosporins, carbapenems)	Weak	Very low
32. In patients with sepsis and an unspecified or possible immediate type penicillin allergy, we suggest to plan penicillin allergy testing and/or a controlled penicillin challenge when the patient has recovered from sepsis	Weak	Very low
III Timing and duration of antibacterial therapy in sepsis		
What is the optimal timing of empirical antibacterial therapy in patients with sepsis?		
33. In patients with sepsis or septic shock, we recommend that the administration of antibacterial treatment should be initiated promptly with health care systems working to reduce that time to as short a duration as feasible	Strong	Low
What is the optimal duration of antibacterial treatment for sepsis?		
34. For treatment duration of sepsis due to CAP, UTI, SSSI and of sepsis due to *S. aureus* infection, we refer to other guidelines [67, 109, 110, 116–118]		
35. We recommend source control interventions when possible to support antibacterial treatment in patients with sepsis	Strong	Low
36. We recommend that a 4-day course of antibacterial treatment is appropriate for patients with sepsis due to intra-abdominal infections following effective source control and with favourable clinical response	Strong	Moderate
37. We suggest that shorter courses of antibacterial treatment (up to 3 days) are appropriate in patients with sepsis and cholangitis following adequate drainage of the biliary tree	Weak	Very low
38. We recommend that an antibacterial treatment duration of 7 days is adequate for most patients with sepsis due to VAP	Strong	Moderate
39. We suggest that an antibacterial treatment duration of 7 days is adequate for most patients with sepsis due to HAP	Weak	Very low
40. We suggest that an antibacterial treatment duration of 7 days at maximum is adequate for most patients with sepsis due to suspected CVC infection with gram-negative pathogens following removal of the CVC and with favourable clinical response	Weak	Very low
41. We suggest that an antibacterial treatment duration of 0 to 7 days is adequate for most patients with sepsis due to suspected CVC infection with CNS or enterococci following removal of the CVC and with favourable clinical response	Weak	GPS
42. We suggest that an antibacterial treatment duration of 7 days is adequate for sepsis and septic shock without a clear focus in most patients with favourable clinical response	Weak	Low
43. We recommend daily assessment for the need of antibacterial therapy in patients with sepsis and to discontinue therapy when during follow-up there is lack of clinical or microbiological evidence of infection	Strong	GPS
44. We suggest that procalcitonin levels are used to support shortening the duration of antibacterial therapy in patients with sepsis if optimal duration of antibiotic therapy is unclear	Weak	Moderate
45. We recommend to consider antibiotic de-escalation (resulting in smaller spectrum antibiotics) in all patients on antibiotics for sepsis on a daily basis and based on pathogen identification, sensitivities and risk of adverse events	Strong	Very low
46. We recommend to stop empirical aminoglycoside therapy within a maximum of two days	Strong	Low
47. We recommend to switch systemic antibiotic therapy from intravenous to oral antibiotic therapy after 48–72 h on the basis of the clinical condition and when oral treatment is feasible	Strong	Very low
IV Pharmacokinetic and pharmacodynamic considerations in sepsis		
In patients with sepsis, should we recommend pharmacokinetic/pharmacodynamic dosing optimization for empirical antibacterial therapy?		
48. In patients with sepsis, we suggest that dosing strategies of antibacterial therapy be optimized based on accepted pharmacokinetic / pharmacodynamic principles and specific drug properties (Additional file 1: Table S3)	Weak	Low
49. In patients with sepsis we recommend prolonged or continuous^d^ infusion of piperacillin-tazobactam and carbapenems	Strong	High
50. In patients with sepsis we suggest prolonged or continuous^d^ infusion of other beta-lactam antibiotics than piperacillin-tazobactam and carbapenems	Weak	Low
51. In patients with sepsis, we recommend direct therapeutic drug monitoring (including either mid-dosing or both peak and through levels) during aminoglycoside treatment in patients with sepsis and septic shock	Strong	GPS
52. In patients with sepsis, we recommend therapeutic drug monitoring during vancomycin treatment in patients with sepsis and septic shock	Strong	GPS
53. In patients with sepsis, we suggest therapeutic drug monitoring when there are concerns on target attainment of other antibacterial drugs than aminoglycoside and vancomycin (e.g., extreme body weight, augmented or decreased renal clearance, hypoalbuminemia)	Weak	GPS
54. In patients with sepsis, we suggest continuous^d^ infusion of vancomycin	Weak	GPS
55. In patients with sepsis in whom ciprofloxacin is indicated, we suggest empirical ciprofloxacin three times daily 400 mg iv	Weak	GPS

^a^We defined double active antibacterial therapy as treatment with two classes of antibiotics, both targeting the known or suspected causing pathogen(s) (e.g., ceftriaxone and an aminoglycoside to target gram-negative pathogens) and with the specific purpose to accelerate pathogen clearance rather than to broaden antimicrobial coverage. Also frequently referred to as combination antibiotic therapy. Of note, the use of two antibiotics for the increased likelihood of covering the causing agent (broadening the spectrum), or for covering multiple causing agents (e.g., aerobic and anaerobic bacteria) was not included in the definition of double active therapy

^b^Recommendations for sepsis due to suspected long-term CVC’s were not included in this guideline

^c^3GC may be given in high dose for more optimal PK/PD for *S. aureus* infections in accordance to EUCAST

^d^Continuous infusion includes one intermittent dose as a loading dose

Textbox 1: What is new since the Dutch 2010 SWAB guidelines were published?

**Table:** 

• One important revision is the distinction between low, increased and high risk of infection with Enterobacterales resistant to third generation cephalosporins (3GRC-E) to guide the choice of empirical therapy. 3GCR-E is often used as a proxy for ESBL-production. The committee recommends covering 3GCR-E in patients if prior (1-year) culture revealed 3GCR-E. In patients without prior (1-year) cultures showing 3GCR-E the decision to empirically cover 3GCR-E should be made on an individual patient basis, taking into account multiple risk factors
• The choice of empirical antibacterial treatment of sepsis is dictated by the likelihood of involvement of a resistant causative pathogen, by the desire to prevent overuse of reserve antibiotics from a stewardship perspective and by risks of toxicity and other potential adverse events for the patient. Strong recommendations on the best empirical treatment in sepsis based on the currently available literature cannot be given since only subtle differences between strategies on clinical outcomes are found in studies that were also frequently not generalizable to the Dutch clinical setting. Every strategy has advantages and disadvantages depending on the mentioned perspectives (resistance epidemiology, pharmacokinetic/pharmacodynamic (PK/PD) properties, antibiotic stewardship, adverse events). Consequently, the committee provided pragmatic suggestions for empirical treatment choices in patients with sepsis based on current evidence, reported resistance rates nationally, the antibiotic stewardship perspective and risk of adverse events
• In patients with sepsis, we generally recommend using a beta-lactam antibiotic covering the most likely involved pathogens. Also, we recommend covering pathogens in prior (1-year) relevant cultures in general. We added suggestions on empirical therapy in case *Pseudomonas aeruginosa*, *Staphylococcus aureus* and *Enterococcus* spp are considered
• We provided new suggestions for empirical therapy in patients with sepsis and a reported penicillin allergy
• Regarding the duration of therapy, we generally recommended shorter treatment durations of patients with sepsis than the previous guidelines. The committee also underscores the responsibility of clinicians to de-escalate antibacterial therapy in patients with sepsis. Due to toxicity concerns, we strongly recommend stopping empirical aminoglycoside treatment after 2 days
• Among recommendations on PK/PD considerations in patients with sepsis, the committee strongly recommends continuous or prolonged infusion of piperacillin-tazobactam and meropenem based on high quality evidence. Therapeutic drug monitoring is recommended for all patients on aminoglycoside and vancomycin treatment

## Conclusions

We described the most important findings and recommendations of our multidisciplinary guideline committee for the 2020 SWAB sepsis guidelines. We formulated 55 recommendations on the antibacterial management of sepsis in adults in total. One crucial revision is the distinction between low, increased and high risk of infection with Enterobacterales resistant to third generation cephalosporins (3GRC-E) to guide the choice of empirical therapy. Other new topics included empirical antibacterial therapy in patients with a reported penicillin allergy and the role of pharmacokinetics and pharmacodynamics to guide dosing in sepsis.

## Supplementary Information

**Additional file 1. sTable 1**. Alternative empirical treatment strategies in sepsis and low estimated risk of involvement of 3GCR-E.** sTable 2**. Alternative empirical treatment strategies in sepsis and increased or high estimated risk of involvement of 3GCR-E.** sTable 3**. Recommended iv doses of empirical antibacterial treatment for sepsis.

## Data Availability

Literature searches are available from the corresponding author upon request.
