# Supplementary material for: The 2021 Dutch Working Party on Antibiotic Policy (SWAB) guidelines for empirical antibacterial therapy of sepsis in adults
Source: BMC Infect Dis. 2022 Aug 11;22:687. doi: 10.1186/s12879-022-07653-3 (PMC9373543; doi:10.1186/s12879-022-07653-3)
Supplement: Supplementary file 1 — Additional file 1. sTable 1. Alternative empirical treatment strategies in sepsis and low estimated risk of involvement of 3GCR-E. sTable 2. Alternative empirical treatment strategies in sepsis and increased or high estimated risk of involvement of 3GCR-E. sTable 3. Recommended iv doses of empirical antibacterial treatment for sepsis. [file 12879_2022_7653_MOESM1_ESM.docx]

**sTable 1. Alternative empirical treatment strategies in sepsis and low estimated risk of involvement of 3GCR-E**

| **Source** | **Choice** | **Empirical treatment strategy** | **Advantages** | **Disadvantages** | **Note** |
| --- | --- | --- | --- | --- | --- |
| **Unknown** | 1^st^ | 3GC | Relatively small spectrum  Low risk of adverse events  Only beta-lactam component of therapy | Potentially suboptimal *S. aureus* PK/PD | High dose 3GC optional when there is a higher likelihood of *S. aureus* involvement |
|  | Alternative | Piperacillin-tazobactam | Spectrum includes *E. faecalis* anaerobic bacteria and *P. aeruginosa*  Only beta-lactam component of therapy | Broader antibacterial spectrum compared to 1^st^ choice  Prolonged or continuous infusion strongly recommended | Optional when there is a higher likelihood of anaerobic, *P. aeruginosa* or enterococcal involvement |
|  | Alternative | 2GC plus high dose ciprofloxacin or an aminoglycoside | Spectrum includes *P. aeruginosa* Potentially better *S. aureus* PK/PD of beta-lactam component | Potentially less optimal Enterobacterales PK/PD of beta-lactam component  Potentially higher risk of adverse events compared to 1^st^ choice  TDM and max 2 day treatment for aminoglycoside | Optional when there is a higher likelihood of *S. aureus* infection or *P. aeruginosa* involvement |
| **HAP or VAP** | 1^st^ | 3GC plus high dose ciprofloxacin | Spectrum includes *P. aeruginosa*. Potentially better Enterobacterales PK/PD of beta-lactam component (compared to 2GC) | No anaerobic coverage. Potentially less optimal *S. aureus* PK/PD of beta-lactam component (compared to 2GC) |  |
|  | 1^st^ | Piperacillin-tazobactam | Spectrum includes *P. aeruginosa* and anaerobic bacteria  Only beta-lactam component of therapy | Broader antibacterial spectrum |  |
|  | 1^st^ | 2GC plus high dose ciprofloxacin | Spectrum includes *P. aeruginosa*. Potentially better *S. aureus* PK/PD of beta-lactam component (compared to 3GC) | No anaerobic coverage. Potentially less optimal Enterobacterales PK/PD of beta-lactam component (compared to 3GC) |  |
| **Cholangitis** | 1st | 3GC | Relatively small spectrum  Low risk of adverse events  Only beta-lactam component of therapy | No anaerobic or enterococcal coverage. | Addition of metronidazole in patients with sepsis due to cholangitis who have biliary-enteric anastomosis |
|  | Alternative | Piperacillin-tazobactam | Spectrum includes *E. faecalis*, anaerobic bacteria and *P. aeruginosa* | Broader antibacterial spectrum  Prolonged or continuous infusion strongly recommended | Optional when there is a higher likelihood of anaerobic, enterococcal or *P. aeruginosa* involvement  Alternative treatment option to 3GC plus metronidazole in patients with sepsis due to cholangitis who have biliary-enteric anastomosis |
|  | Alternative | 2GC/3GC plus high dose ciprofloxacin or an aminoglycoside | Spectrum includes *P. aeruginosa* | No anaerobic coverage  Potentially higher risk of adverse events  TDM and max 2 day treatment for aminoglycoside | Optional when there is a higher likelihood of *P. aeruginosa* involvement  Addition of metronidazole in patients with sepsis due to cholangitis who have biliary-enteric anastomosis |
| **Intra-abdominal infection** | 1^st^ | 3GC plus metronidazole | Relatively small spectrum  Low risk of adverse events  Only beta-lactam component of therapy |  |  |
|  | Alternative | Piperacillin-tazobactam | Spectrum includes *E. faecalis* and *P. aeruginosa* | Broader antibacterial spectrum  Prolonged or continuous infusion strongly recommended | Optional when there is a higher likelihood of *P. aeruginosa* or enterococcal involvement |
| **CVC infection**  Multiple equivalent treatment options | 1^st^ | High dose 3GC plus high dose ciprofloxacin or an aminoglycoside | Spectrum includes *P. aeruginosa*  Potentially better *S. aureus* PK/PD (compared to regular 3GC dose) | Risk of adverse events  TDM and max 2 day treatment for aminoglycoside |  |
|  | 1^st^ | Piperacillin-tazobactam | Spectrum includes *E. faecalis* and *P. aeruginosa* | Prolonged or continuous infusion strongly recommended |  |
|  | 1^st^ | 2GC plus high dose ciprofloxacin or an aminoglycoside | Spectrum includes *P. aeruginosa* Potentially better *S. aureus* PK/PD of beta-lactam component (compared to regular 3GC dose) | Potentially less optimal Enterobacterales PK/PD of beta-lactam component (compared to 3GC)  Risk of adverse events  TDM and max 2 day treatment for aminoglycoside |  |
|  | Alternative | Flucloxacillin plus high dose ciprofloxacin or aminoglycoside | Optimal *S. aureus* therapy | No beta-lactam treatment of gram-negative pathogens  Higher risk of adverse events  TDM and max 2 day treatment for aminoglycoside | Optional, especially when there is a high likelihood of *S. aureus* involvement |
|  | Alternative | Vancomycin plus gram-negative antibacterial treatment | Spectrum includes coagulase-negative Staphylococci, Enterococci | Potentially less optimal *S. aureus* treatment | Optional when there is a high likelihood of enterococcal involvement or when the patient has intravascular prosthetic material at risk of secondary infection with low virulent gram-positive pathogens |

**sTable 2. Alternative empirical treatment strategies in sepsis and increased or high estimated risk of involvement of 3GCR-E**

| **Estimated risk of involvement of 3GCR-E** | **Choice** | **Empirical treatment strategy** | **Advantages** | **Disadvantages** | **Note** |
| --- | --- | --- | --- | --- | --- |
| **Increased risk** | 1^st^ | 2GC/3GC plus an aminoglycoside (plus metronidazole when applicable) | Carbapenem-sparing  Fluoroquinolone-sparing | Potentially higher risk of adverse events compared to other choices  TDM and max 2 day treatment for aminoglycoside  Approximately 1/3^rd^ of ESBL-producing Enterobacterales is resistant to aminoglycosides | Equivalent treatment option when there is no known renal insufficiency |
|  | 1^st^ | Meropenem or imipenem | Only beta-lactam component of sepsis therapy  Potentially lower toxicity profile, especially in case of renal insufficiency | Very broad-spectrum | Equivalent treatment option |
|  | Alternative | 2GC/3GC plus high dose ciprofloxacin (plus metronidazole when applicable) | Carbapenem-sparing  Aminoglycoside-sparing | Approximately 2/3^rd^ of ESBL-producing Enterobacterales is ciprofloxacin-resistant  Risk of adverse events | Optional when local ciprofloxacin resistance allows its empirical use |
| **High risk** | 1^st^ | Meropenem or imipenem | Only beta-lactam component of sepsis therapy  Potentially lower toxicity profile, especially in case of renal insufficiency | Very broad-spectrum | Preferred treatment option, especially when there was previous resistance to aminoglycosides or ciprofloxacin or risk of toxicity (e.g. known renal insufficiency) |
|  | Alternative | 2GC/3GC plus an aminoglycoside (plus metronidazole when applicable) | Carbapenem-sparing, Fluoroquinolone-sparing | Potentially higher risk of adverse events compared to other choices  TDM and max 2 day treatment for aminoglycoside  Approximately 1/3^rd^ of ESBL-producing Enterobacterales is resistant to aminoglycosides | Optional when local resistance epidemiology allows, when there is no sepsis (yet) and/or when the previously cultured 3GCR-E was susceptible |
|  | Alternative | 2GC/3GC plus high dose ciprofloxacin (plus metronidazole when applicable) | Carbapenem-sparing  Aminoglycoside-sparing | Nationally, approximately 2/3^rd^ of ESBL-producing Enterobacterales is ciprofloxacin-resistant | Optional when local ciprofloxacin resistance allows its use and/or when previously cultured 3GCR-E was susceptible |
|  | Alternative | Piperacillin-tazobactam | Carbapenem-sparing  Fluoroquinolone-sparing  Aminoglycoside-sparing | Likely inferior in ESBL-producing 3GCR-E sepsis | Only optional when the previously cultured 3GCR-E did not produce ESBL and was susceptible to piperacillin-tazobactam |

**sTable 3. Recommended iv doses of empirical antibacterial treatment for sepsis**

| **Antibacterial agent** | **Intermittent dosing (<60 min infusion)** | **Prolonged dosing (3-5 hour infusion)** | **Continuous infusion + loading dose** | **Remarks** |
| --- | --- | --- | --- | --- |
| Benzylpenicillin | 6x 1 million IU | 6x 1 million IU | 6 million IU + 1 million IU loading dose | Higher doses optional up to 24 million IU per 24h* |
| Amoxicillin | 6x 1000 mg | 6x 1000 mg | 6000 mg + 1000 mg loading dose | Higher doses optional up to 12000 mg per 24h* |
| Flucloxacillin | 6x 1000 mg | 6x 1000 mg | 6000 mg + 1000 mg loading dose | Higher doses optional up to 12000 mg per 24h* |
| Amoxicillin-clavulanic acid | 4x 1200 mg | 4x 1200 mg | N.a. |  |
| Piperacillin-tazobactam | 4x 4500 mg | 4x 4500 mg | 18000 mg + 4500 mg loading dose | 3x 4500 mg when *Pseudomonas* is not involved* |
| Cefazolin | 3x 1000 mg | 3x 1000 mg | 3000 mg + 1000 mg loading dose | Higher doses optional up to 6000 mg per 24h* |
| Cefuroxime | 3x 1500 mg | 3x 1500 mg | 4500 mg + 1500 mg loading dose |  |
| Ceftriaxone | 1x2000 mg | 1x 2000 mg | 2000 mg + 2000 mg loading dose | 2x 2000 mg when *S. aureus* is involved* |
| Ceftazidime | 3x 2000 mg | 3x 2000 mg (3 hour infusion) | 6000 mg + 2000 mg loading dose | 3x 1000 mg or 3000 mg per 24h + 1000 mg loading dose optional when *Pseudomonas* is not involved |
| Imipenem | 4x 1000 mg | 4x 1000 mg | 4000 mg + 1000 mg loading dose | 4x 500 mg optional when *Pseudomonas* is not involved |
| Meropenem | 3x 1000 mg | 3x 1000 mg (3 hour infusion) | 3000 mg + 1000 mg loading dose | Higher doses optional up to 6000 mg per 24h* |
| Ciprofloxacin | 3x 400 mg | N.a | N.a. | 2x 400 mg when *Pseudomonas* is not involved* |
| Gentamicin | 1x 5 mg/kg** | N.a. | N.a. | 1x 6-7 mg/kg may be indicated in sepsis due to Enterobacterales *  Adjusted for adjusted bodyweight**  Immediate TDM recommended***  Should be given in combination with other antibacterial therapy, generally a beta-lactam agent. Gentamicin may be a suboptimal choice for *P. aeruginosa* based on PK/PD models**** |
| Tobramycin | 1x 5 mg/kg** | N.a. | N.a. | 1x 6-7 mg/kg may be indicated in sepsis due to Enterobacterales or *Pseudomonas**  Adjusted for adjusted body weight**  Immediate TDM recommended***  Should be given in combination with other antibacterial therapy, generally a beta-lactam agent.**** |
| Vancomycin | 2-3x 15-20 mg/kg + 25-30 mg/kg loading dose | N.a. | 30-40 mg/kg + 15-20 mg/kg loading dose | Adjusted for adjusted body weight**  TDM recommended*** |
| Metronidazole | 3x 500 mg | N.a. | N.a. |  |
| Trimethoprim-sulfamethoxazole | 2x 960 mg | N.a. | N.a. | Higher doses optional* |

* See also EUCAST dosing table (<http://www.eucast.org/>) for guidance on which pathogens may require higher dosing and other relevant guidelines for infections that require other dosages. In case of higher 24h doses a higher loading dose is indicated (i.e. one intermittent dose)

** Adjusted for adjusted body weight (ideal body weight + 0,4*(true body weight – ideal body weight. Ideal body weight: man: 50 kg + 0,9 * (cm > 150 cm); woman: 45 kg + 0,9 * (cm > 150 cm). See <https://tdm-monografie.org/>

*** See <https://tdm-monografie.org/>

**** Since 2020 EUCAST doesn’t provide formal breakpoints for aminoglycosides in systemic infection (excluding UTI) with Enterobacterales, *Pseudomonas*, *Acinetobacter* and *Staphylococcus* species anymore. EUCAST recommends that in systemic infections with these species, the aminoglycoside must be supported by other active therapy. In addition, there are no breakpoints for gentamicin in any infection with *Pseudomonas* species anymore. See EUCAST clinical breakpoints table and http://www.eucast.org/guidance_documents/.
